# Supplementary material for: Fragmentation in turbulence by small eddies
Source: Nat Commun. 2022 Jan 25;13:469. doi: 10.1038/s41467-022-28092-3 (PMC8789894; doi:10.1038/s41467-022-28092-3)
Supplement: Supplementary file 1 — Supplementary Information [file 41467_2022_28092_MOESM1_ESM.pdf]

Supplementary Information – Fragmentation in turbulence by small eddies

Yinghe Qi, Shiyong Tan, Noah Corbitt, Carl Urbanik,
Ashwanth K. R. Salibindla, Rui Ni*

Department of Mechanical Engineering, Johns Hopkins University, Baltimore, MD-21218, USA

1 The Local Weber Number (HIT versus Vortex Ring)

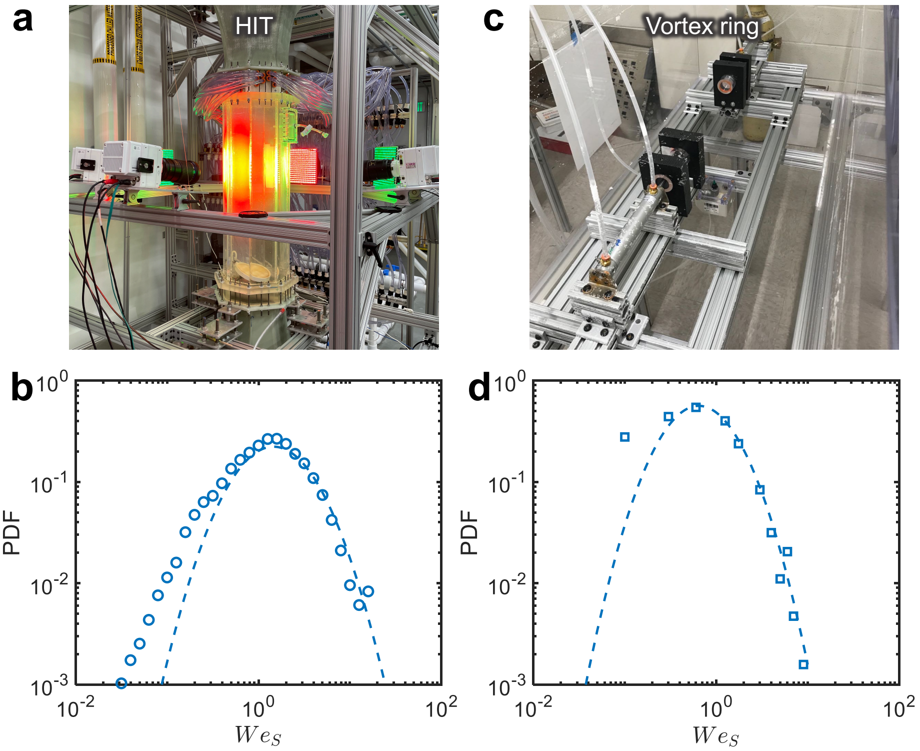

Figure S1: (a) Picture of the vertical water tunnel (V-ONSET) that generates turbulence that is nearly homogeneous and isotropic (HIT); (b) The distribution of the local We around breaking bubbles in (a); (c) Picture of the vortex-ring-collision setup; (d) The distribution of the local We around the secondary breaking bubbles in (c).

One may wonder if the secondary bubble breakup in the flow produced by the vortex-ring collision can be directly used to test the key hypothesis in the KH framework, which is widely believed to apply only to the homogeneous and isotropic turbulence.

The key emphasis in the KH framework is the definition of the Weber number (We= $\rho u^2 D/\sigma$) and its key role in bubble/drop breakup. Both Kolmogorov and Hinze started with homogeneous and isotropic turbulence (HIT) because u^2 can be estimated based on the second-order structure function [2]. But the framework was not limited to HIT. In fact, Kolmogorov and Hinze themselves tried to generalize the framework to other types of turbulence, e.g. oil emulsion in a pipe [1]. In this case, u^2 and the associated local We become spatially and temporally varying, and the breakup is determined by the local We. As long as the correct u^2 based on the local flow condition is used, the KH framework should still be applied.

To link the dynamics of the bubble breakup in two seemingly different turbulent flows, we compare the distribution of the local Weber number around individual breaking bubbles in the vortex-ring-collision setup (Fig. S1c) against those in the fully developed, statistically stationary, homogeneous and isotropic turbulence generated by a vertical water tunnel (the V-ONSET) (Fig. S1a) [4, 5]. The local Weber number was calculated based on the rate-of-strain tensor We_S in both experiments using the same method.

The PDFs of We_S for both setups are shown in the bottom row of Fig. S1. In addition to the data, we can also predict this PDF, i.e. $P(\mathrm{We})$, based on the definition of the local We, i.e. $\mathrm{We}{=}\rho(\epsilon_e D)^{2/3}D/\sigma$. The log-normal distribution of ϵ_e, i.e. $P(\epsilon_e)$, can be determined from Eq. 5 in the main text. The PDF of the local Weber number $P(\mathrm{We})$ can then be calculated based $P(\mathrm{We})d\mathrm{We}{=}P(\epsilon_e)d\epsilon_e$. The predicted result is shown as the dashed line in Fig. S1d. For details, one can refer to our previous study [5]. The nice agreement between the data and the dashed line suggests that the turbulence near breaking secondary bubbles is intermittent and close to HIT.

2 Vortex ring collision

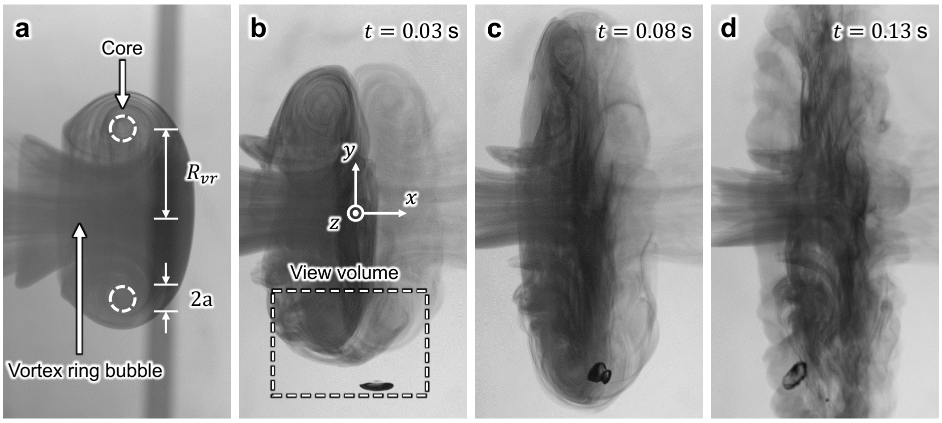

Figure S2: Dye visualization of (a) the left vortex ring (side view) with the core size a and the ring radius R_{vr} marked; (b-d) two vortex rings after collision at $t = 0.03$, 0.08 and 0.13 s, where $t = 0$ is the collision moment the definition of which can be found in Supplementary Fig. S3(b). The cascade process from a large intact vortical structure to a turbulent cloud can be seen. In panel (b), the coordinates and the view volume for our 3D measurement are marked for reference.

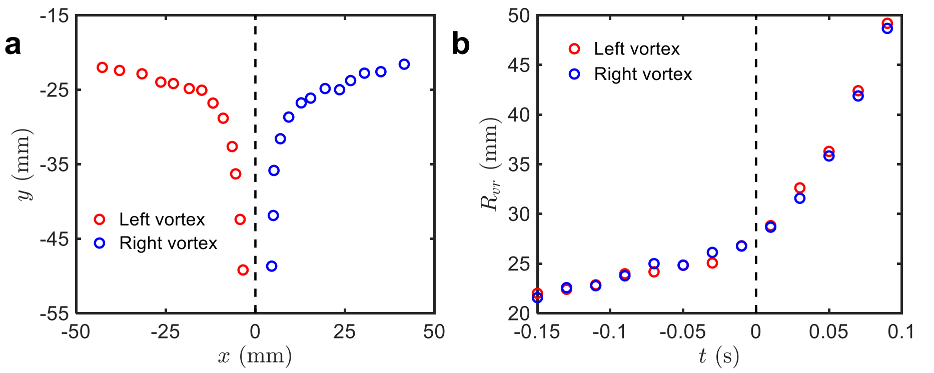

Figure S3: (a) Trajectories of the cores of both vortex rings in the xy plane where $z = 0$. The dashed line marks the collision plane ($x = 0$). (b) Vortex ring radii (R_{vr}) over time. The dashed line indicates the moment when the vortex ring collision happens.

Supplementary Fig. S2 shows the side view of the collision process between the two vortex rings. We rely on the model by Sullivan et al. [7] to estimate important parameters of the vortex ring when it just

gets expelled out of the cylinder exit. For example, the vortex ring radius R_{vr}, which is defined as the radius of the ring structure, can be calculated following:

$$R_{vr} = \left(\frac{3(D_0/2)^2 L_s}{4\gamma} \right)^{1/3} \tag{1}$$

where γ is the eccentricity of the vortex ring within the x-y plane. Based on $\gamma = 0.6$ determined from Supplementary Fig. S2a, we estimate the vortex ring radius to be $R_{vr} = 27.4$ mm. The core size of the vortex ring, where the vorticity is strongest, is linked to the piston stroke time (T_s) and the fluid viscosity (ν):

$$a = \sqrt{4\nu T_s} \tag{2}$$

which leads to $a = 0.93$ mm. The circulation of the vortex ring Γ follows:

$$\Gamma = \frac{(D_0/2)^2 L_s^2}{R_{vr}^2 T_s} \tag{3}$$

By taking into all parameters from our experiments, Γ is roughly 0.01 $\mathrm{m^2/s}$. Finally, the initial translational velocity of the vortex ring can be estimated by using:

$$V_0 = \frac{\Gamma}{4\pi R_{vr}} \left(\ln\left(8R_{vr}/a\right) - 0.558 \right) \tag{4}$$

Substituting Γ into this equation yields $V_0 = 0.15$ m/s.

In experiments, we track the bottom portion of the two rings until they meet and collide at the plane of $x = 0$. Supplementary Fig. S3a shows the time trace of the central locations of these two bottom vortices, from which the horizontal velocity (along x-axis) of both rings can be estimated, i.e. 0.13 m/s. Compared to V_0 of 0.15 m/s predicted by the model [7], the measured velocity appears to be very close but slightly smaller because the vortex rings slow down as they entrain low-momentum fresh water when they move closer to each other [7]. Once the two rings collide, their horizontal velocities quickly decrease to almost zero and their vertical motions (along the y-axis) accelerate. The core tracks of both the left and right vortex rings in Supplementary Fig. S3a seem to be symmetric about the collision plane, suggesting that the experimental control is precise enough that the collision is indeed head-on and symmetric, as designed.

Based on the bottom vortex core locations, the vortex ring radius R_{vr} can be determined as a function of time, which is shown in Supplementary Fig. S3b. R_{vr} starts at about 22 mm, which is close to the predicted value of 27.4 mm based on the model [7]. Consistent with Supplementary Fig. S3a, the vortex ring radii get expanded slowly before the collision and rapidly after. Therefore, we define the collision time t_0 by the transition moment of the expansion rate, as shown by the dashed line in Supplementary Fig. S3b.

3 Particle tracking

For the carrier phase, 60-μm-diameter neutrally-buoyant particles were used as tracers. To view them clearly on cameras, laser illumination is typically favorable because it helps to select only the particles in the volume of interest. However, for flows that contain bubbles, the interface between the two phases is highly reflective, leaving shadows and light stripes in the view. This problem can severely impact the ability to acquire accurate tracer data around bubbles. This issue is resolved using the shadow imaging. Compared with all other methods, the back illumination can achieve a clear, sharp shadow projection of both bubbles and tracers.

Tracer particles, after being segmented from the raw images, were tracked using our in-house OpenLPT method [8]. This code has a built-in linear-fit check to ensure correct particle trajectories. Any tracks with strong position oscillation were categorized as wrong tracks and deleted in this step. However, this step can potentially cause broken trajectories. OpenLPT splices these broken segments back by performing backward tracking after the first forward tracking. This will connect and extend the existing track segments.

Sample 3D particle tracks, color coded with the instantaneous particle velocity magnitude $|\boldsymbol{u}^p|$, are shown in Fig. S4a. Particles with velocity magnitude ranging from close to zero in the background to more than 0.6 m/s between two rings can all be tracked successfully by our in-house tracking method.

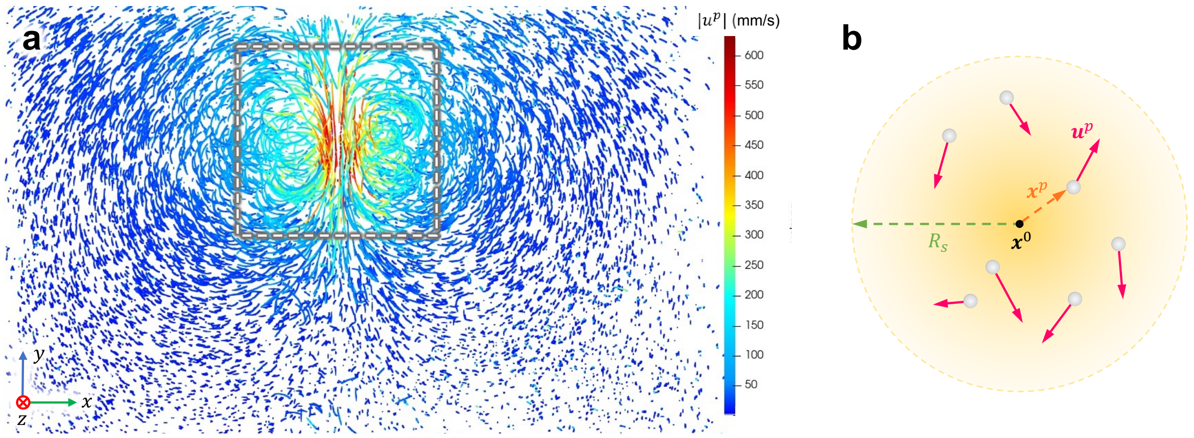

Figure S4: (a) Sample particle trajectories from $t = 0.06$ s to $t = 0.10$ s generated by the OpenLPT method. The dashed box indicates the volume that was used to calculate the velocity structure function; (b) The schematic to show how the vorticity is calculated for any points of interest $\boldsymbol{x}^0$ by utilizing all the particle within the search radius of R_s from $\boldsymbol{x}^0$.

4 Structure function and velocity gradients

4.1 Structure function

Instead of having velocity on a grid, the flow velocity is available at locations where we have particles. For the structure function calculations, pairs of particles with separation $\boldsymbol{r}$ have to be identified first, from which the second-order Eulerian structure functions, including both the longitudinal ($D_{LL}(r) = \langle [u_{\|}(\boldsymbol{x}+\boldsymbol{r}) - u_{\|}(\boldsymbol{x})]^2 \rangle$) and transverse ($D_{NN}(r) = \langle [u_{\perp}(\boldsymbol{x}+\boldsymbol{r}) - u_{\perp}(\boldsymbol{x})]^2 \rangle$) components, can be calculated. $u_{\|}$ and $u_{\perp}$ represent the tracer velocity projected to directions that are parallel and perpendicular to the separation direction $\boldsymbol{r}$ between a pair of particles located at $\boldsymbol{x} + \boldsymbol{r}$ and $\boldsymbol{x}$, respectively. Many pairs of particles were used to ensure a converged ensemble average ($\langle ... \rangle$).

The structure function was calculated in the dashed box, as shown in Fig. S4. This box is about $30 \times 30 \times 30$ mm^3 and covering the entire region where the two vortices were located. This box continues to move down with the vortex rings following the same speed to ensure that the statistics collected is not contaminated by other regions and roughly the same number of velocity pairs are used.

4.2 Velocity gradients

The local flow velocity gradients can be determined from the 3D particle tracking results. At any location $\boldsymbol{x}^0$, the velocity gradient tensor A_{ij} at this point is determined based on the velocity $\boldsymbol{u}^p$ of n tracer particles ($p = 1, 2, ..., n$) around $\boldsymbol{x}^0$ within a search radius of R_s. By using the Taylor expansion, the velocity can be decomposed into the first two leading terms:

$$\boldsymbol{u}^p(\boldsymbol{x}^0 + \boldsymbol{x}^p) \approx \langle \boldsymbol{u} \rangle(\boldsymbol{x}^0) + A_{ij}(\boldsymbol{x}^0)\boldsymbol{x}^p \tag{5}$$

where $\boldsymbol{x}^p$ is the vector pointing to the position of tracer particle from $\boldsymbol{x}^0$, and $\langle \boldsymbol{u} \rangle = \sum_{p=1}^{n} \boldsymbol{u}^p/n$ represents the local mean flow. A unique A_{ij} can be determined if there are only four particles within the search radius R_s. In practice, we need more particles to perform the least-square fit by seeking minimum squared residuals $\sum_p [\boldsymbol{u}^p - \overline{\boldsymbol{u}} - A_{ij}\boldsymbol{x}^p]$ to get converged results [5]. Once A_{ij} is determined, the strain rate and the vorticity can be easily calculated from the rate of strain tensor $S_{ij} = (A_{ij} + A_{ji})/2$ and rotation tensor $\Omega_{ij} = (A_{ij} - A_{ji})/2$, respectively. This method of calculating the velocity gradient tensor has been employed and validated in Ref. [6, 5].

For the vorticity and rate of strain around breaking bubbles, $\boldsymbol{x}^0$ is located at the center of mass of bubbles, and particles within the search radius R_s were included for the calculation. The selection and correction of R_s can be found in Ref. [5]. The concentration of tracers is limited so R_s that we have used is the finest scale that the data can afford. R_s was also kept the same for both the primary and secondary breakups for fair comparisons. In addition, for the 3D iso-surface of vorticity in Fig. 3b, an underlying 3D grid is required. For this case, $\boldsymbol{x}^0$ is selected to be the center of each grid, and we used a constant R_s to calculate the vorticity. The 3D grid spacing is much finer than R_s to achieve a better figure quality.

Admittedly, the finite R_s will result in the filtering effect, indicating that turbulence visualized may not capture all the fine structures beyond our spatial resolution. Nevertheless, even with this filter, the flow structures nearby the secondary breakups are already chaotic, as shown in Fig. 3b, suggesting that the unfiltered flow could only be more turbulent.

5 Separating the primary and secondary breakups

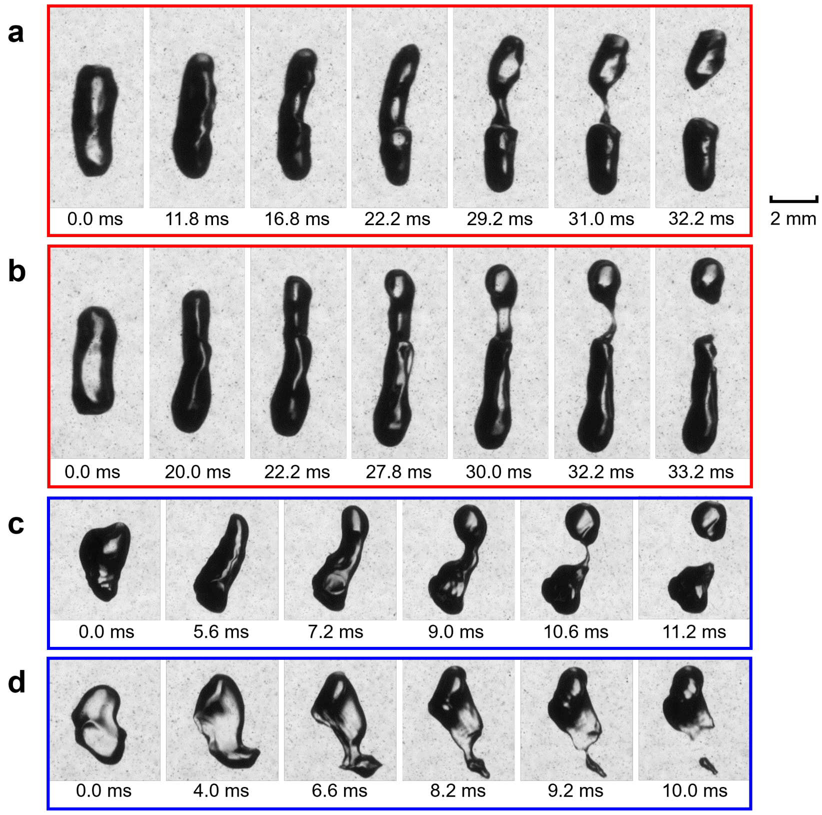

Figure S5: Examples of four bubble breakups, including (a-b) two primary cases (red) and (c-d) two secondary cases (blue)

In the main manuscript, we described the height criterion to separate the primary and secondary breakups. In practice, we also examined this criterion against two other possible methods. The first method is a simple visual check. Additional two examples of the primary and secondary breakups are shown in Fig. S5. Consistent with the example shown in Fig. 2a, the primary breakup is always along one direction, and the secondary breakup is more chaotic with no clear deformation direction. The second method is time. Based on the flow measurement, turbulence began to emerge at about t=0.1 s. Most primary breakups took place before this time, whereas most the secondary breakups occurred after.

In Fig. S6, red circles and blue squares mark the locations of all primary and secondary breakups, respectively, identified by all the three methods. The dashed line indicates the height criterion used in the main manuscript. It can be seen that it separates the two breakup modes successfully. In addition, magenta triangles mark the ambiguous cases that cannot satisfy all three criteria. They account for roughly 15% of the total dataset, and including or excluding them will not change the conclusion.

In addition to the point cloud, we also added the vorticity contour at t=0.05 s and t=0.16 s in the background to show the background flows near the primary and secondary breakups. It can be seen that all primary breakups are inside the regions covered by large vortical structures, whereas the secondary breakups are in the turbulent cloud filled with small eddies.

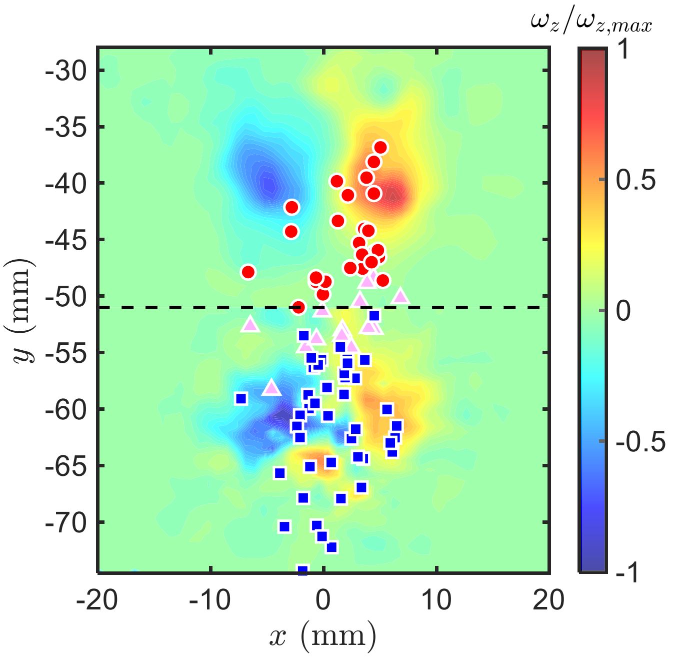

Figure S6: The locations of the primary (red circles) and secondary (blue squares) breakups, along with ambiguous cases (magenta triangles). The black dash line represents the height criterion to separate the primary and secondary breakups. The color map in the background shows the spatial distribution of the normalized vorticity $\omega_z/\omega_{z,max}$ in the xy plane ($z = 0$), where the upper part represents the vorticity field at $t = 0.05$ s while the lower part shows the vorticity field at $t = 0.16$ s.

6 Collision frequency

In this section, we will discuss the bubble-eddy collision rate used in the new model. This rate depends on the collision area ($\sim \pi D^2/4$), the number density of the eddy (n_e), and the eddy velocity ($\langle u_e \rangle$), following the expression of $\omega_c = (\pi/4)D^2 n_e \langle u_e \rangle$ inspired by the gas kinetic theory [3]. $\langle u_e \rangle$ is the mean eddy velocity, which can be expressed as $\langle u_e \rangle \approx \sqrt{2}(\langle \epsilon \rangle D_e)^{1/3}$ based on the inertial range scaling in the Kolmogorov theory [2]. The eddy number density n_e scales with D_e^{-4}. Substituting all the terms into the collision rate immediately yields an expression for ω_c

$$\omega_c = C_1 \frac{\langle \epsilon \rangle^{1/3} D^2}{D_e^{11/3}} \tag{6}$$

where the prefactor C_1 is unknown. In practice, C_1 is determined by fitting against experiments of only one bubble size $D = 3$ mm in Fig. 4f in the main text. The same $C_1 \approx 0.1$ is then used for other bubble sizes. Since C_1 is a constant, it does not affect the overall dependence of the breakup frequency either on the bubble size or on the energy dissipation rate.

Supplementary References

[1] PH Clay. The mechanism of emulsion formation in turbulent flow. *Proceedings of the Section of Sciences*, 43:852–965, 1940.

[2] Andrey Nikolaevich Kolmogorov. The local structure of turbulence in incompressible viscous fluid for very large Reynolds numbers. *Cr Acad. Sci. URSS*, 30:301–305, 1941.

[3] Hean Luo and Hallvard F Svendsen. Theoretical model for drop and bubble breakup in turbulent dispersions. *AIChE Journal*, 42(5):1225–1233, 1996.

[4] Ashik Ullah Mohammad Masuk, Ashwanth Salibindla, Shiyong Tan, and Rui Ni. V-ONSET (Vertical Octagonal Noncorrosive Stirred Energetic Turbulence): A vertical water tunnel with a large energy dissipation rate to study bubble/droplet deformation and breakup in strong turbulence. *Review of Scientific Instruments*, 90(8):085105, 2019.

[5] Ashik Ullah Mohammad Masuk, Ashwanth KR Salibindla, and Rui Ni. Simultaneous measurements of deforming Hinze-scale bubbles with surrounding turbulence. *Journal of Fluid Mechanics*, 910:A21, 2021.

[6] Alain Pumir, Eberhard Bodenschatz, and Haitao Xu. Tetrahedron deformation and alignment of perceived vorticity and strain in a turbulent flow. *Physics of Fluids*, 25(3):035101, 2013.

[7] Ian S. Sullivan, Joseph J. Niemela, Robert E. Hershberger, Diogo Bolster, and Russell J. Donnelly. Dynamics of thin vortex rings. *Journal of Fluid Mechanics*, 609:319–347, 2008.

[8] Shiyong Tan, Ashwanth Salibindla, Ashik Ullah Mohammad Masuk, and Rui Ni. Introducing OpenLPT: new method of removing ghost particles and high-concentration particle shadow tracking. *Experiments in Fluids*, 61(2):47, 2020.
